# Serum CA125, CA199, estradiol, FSH, IL-6, TNF-a in endometriosis after administration of Tripterygium wilfordii glycosides

**DOI:** 10.5937/jomb0-56632

**Published:** 2025-07-04

**Authors:** Fangdan Xu, Ying Xu, Jianping Qiu

**Affiliations:** 1 The Affiliated Suzhou Hospital of Nanjing Medical University, Department of Obstetrics and Gynecology, Suzhou, Jiangsu Province, China

**Keywords:** serum CA125, CA199, estradiol, FSH, IL-6, TNF-a, endometriosis, inflammatory factor, serum sex hormones, therapeutic effect, Tripterygium wilfordii glycosides, tumour markers, serumski CA125, CA199, estradiol, FSH, IL-6, TNF-a, endometrioza, inflamatorni faktor, serumski hormoni, terapijski efekat, glikozidi Tripterygium wilfordii, tumor markeri

## Abstract

**Background:**

To evaluate the therapeutic effects of Tripterygium wilfordii glycosides on endometriosis (EMs) and their effects on serum sex hormone levels, tumour markers, and inflammatory factors (serum CA125, CA199, Estradiol, FSH, IL-6, TNF-a).

**Methods:**

A total of 108 patients were randomly divided into control, progesterone, and Tripterygium wilfordii glycoside-treated groups, each receiving treatment for three months. The clinical efficacy and serum levels of estradiol, follicle-stimulating hormone, tumour markers (cancer antigen125 and cancer antigen199), and pro-inflammatory cytokines were compared before and after treatment.

**Results:**

The Tripterygium wilfordii glycoside group exhibited a significantly higher clinically effective rate (90.74% versus 75.93%) and more significant reductions in estradiol, follicle-stimulating hormone, cancer antigen125, cancer antigen199, IL-6, TNF-a, and HSCRP levels than the control group.

**Conclusions:**

Tripterygium glycosides exhibit significant clinical efficacy in treating patients with EMS by significantly improving serum sex hormone levels, reducing tumour marker levels, alleviating inflammatory reactions, and exerting minimal toxic side effects.

## Abstract (sr)

**Uvod:**

Procena terapijskih efekata glikozida Tripterygium wilfordii na endometriozu (EMs) i efekta na nivoe serumski hormona, tumor markere i inflamatorne faktore (serumski CA125, CA199, estradiol, FSH, IL-6, TNF-a).

**Metode:**

Ukupno 108 pacijentkinja je slučajnim odabirom podeljeno u tri grupe: kontrolnu, grupu tretiranu progesteronom i grupu tretiranu glikozidima Tripterygium wilfordii, pri čemu je svaka grupa primala tretman u trajanju od tri meseca. Pre i posle tretmana upoređivani su klinička efikasnost i nivoi seruma estradiola, hormona koji stimuliše folikul, markeri tumora (antigen raka 125 i antigen raka 199) i pro-inflamatorni citokini.

**Rezultati:**

Grupa koja je primala glikozide Tripterygium wilfordii je imala značajno viši klinički efekat (90,74% u odnosu na 75,93%) i značajnije smanjenje nivoa estradiola, hormona koji stimuliše folikul, antigena raka 125, antigena raka 199, IL-6, TNF-a i HS-CRP u poređenju sa kontrolnom grupom.

**Zaključak:**

Glikozidi Tripterygium wilfordii pokazuju značajnu kliničku efikasnost u lečenju pacijentkinja sa EMS poboljšanjem serumski hormona, smanjenjem nivoa markera tumora, ublažavanjem inflamatornih reakcija i minimalnim toksičnim efektima.

## Introduction

A benign gynaecological disease, endometriosis (EMs), involves the growth of endometrial tissue outside the uterine cavity, including the myometrium, the ovaries, and the pelvis. It occurs mainly in women of childbearing age, and its clinical manifestations often include dysmenorrhea, abnormal menstruation, chronic pelvic pain, and infertility. It has emerged as a prevalent and challenging disease in clinical gynaecology [1]. Despite the apparent benign histopathological changes, it has many similarities with invasive tumours. The incidence rate has gradually increased in recent years, showing a trend towards younger patients due to the influence of socioeconomic factors, environment, and cesarean section [2].

Currently, laparoscopic surgery is the primary clinical treatment for EMs with postoperative medication. However, most patients require long-term medication treatment because of the unknown aetiology of EMs, high recurrence rate, and low pregnancy rate in patients with a prognosis. Therefore, selecting drugs that can effectively prevent recurrence without side effects has become a focus of clinical attention [3]. *Tripterygium* glycosides are extracts from the xylem fragments of *Tripterygium* roots, used in traditional Chinese medicine to remove wind and dampness, promote blood circulation, and dredge collaterals. Modern pharmacology has demonstrated anti-inflammatory, antibacterial, antitumour, and immunosuppressive effects. Clinical and animal experiments have confirmed that *Tripterygium* glycosides have a significant reversible inhibitory effect on the endometrium and ovaries [4]. Gestrinone is a commonly used progestogen in clinical practice that can inhibit the growth of the endometrium and ectopic lesions through its anti-gonadotropin and anti-estrogen effects. Here, *Tripterygium* glycosides combined with gestrinone were used to treat patients with EMs, and their effects on serum sex hormone levels, tumour markers, and inflammatory factors were evaluated.

## Materials and methods

### Study subjects

This study included one hundred eight patients with EMs admitted to our hospital between August 2023 and October 2024.

Inclusion Criteria: All patients met the diagnostic criteria for EMs established by the Chinese Medical Association’s Obstetrics and Gynecology Department’s »Guidelines for the Diagnosis and Treatment of EMs« [5]. All patients were diagnosed using laboratory tests, ultrasound, and laparoscopy; aged 20–50 years, with no childbearing in the last two years; first-time diagnosis of EMs and no previous treatment; chocolate cyst <6 cm; informed consent and cooperation with treatment from patients and their families; and approval of this study by the hospital’s ethics committee.

Exclusion Criteria: Patients with uterine fibroids and pelvic infections; patients with a history of steroid hormone therapy within six months; allergy to the study drugs; severe hepatic, renal, or systemic immune system dysfunction; pregnant and lactating women; and patients with mental disorders.

The patients were divided into the control and treatment groups, with 54 patients in each group. The average age was 32.87±3.25 years, with a disease duration of 4.68±0.79 years. The menstrual cycle lasted 26-30 days, averaging 27.81±4.02 days. There were 38 stage III and 16 stage IV patients. The average age in the control group was 33.75±3.49 years, with a disease duration of 5.16±0.68 years and a menstrual cycle of 27–30 days, with an average of 27.02±5.15 days. The general data of the two groups were comparable (P>0.05) even though there were 35 patients with stage III and 19 with stage IV lesions.

### Therapeutic protocols

Patients in the control group were administered oral gestrinone capsules (2.5 mg/capsule, China Resources Zizhu Pharmaceutical Co., Ltd., batch number 20160526) at a dose of 2.5 mg. The first dose was administered on the first day after menstruation, and the subsequent dose was administered three days later, twice a week. Patients in the treatment group were administered *Tripterygium* glycoside tablets (30 mg, Jiangsu Metong Pharmaceutical Co., Ltd., batch number: 20150928) at 20 mg three times a day. After four weeks, the dose was reduced to 10 mg. Both groups were treated for three months as a course of treatment.

### Observational indicators

Clinical efficacy [6] was assessed and grouped as follows:

a) Recovered: After treatment, the patient’s clinical symptoms completely resolved, the pelvic mass disappeared, and laboratory and ultrasound examinations were normal.

b) Significant effect: After treatment, the patient’s clinical symptoms disappeared, the pelvic mass decreased by 1/4, and laboratory and ultrasound examinations were normal.

c) Effective: The patient’s clinical symptoms improved, pelvic mass slightly decreased, and laboratory and ultrasound examinations improved after treatment.

d) Ineffective: No improvement in clinical symptoms and signs, laboratory tests, or ultrasound examinations.

Total effective rate = (recovery + significant effect + effective)/total cases × 100

Serum sex hormone levels: Changes in E) and FS) levels were assessed in both groups before and after treatment using enzyme-linked immunosorbent assay.

Tumour markers: Serum CA125 and CA199 levels were measured in both groups before and after treatment using chemiluminescence.

Pro-inflammatory cytokines: IL-6, TNF-α, and HSCRP levels were measured in both groups before and after treatment using enzyme-linked immunosorbent assay.

Adverse reactions were observed in both groups during the treatment period.

### Statistical methods

Data were compared between the control and experimental groups using a t-test. All count data were expressed as [n (%)] and compared between the two groups using the two-tailed test. The significance level was set at P<0.05 in the Statistical Package for the Social Sciences software version 21.0.

## Results

### Clinical efficacy between the control and Tripterygium glycosides groups

Table 1 shows that the overall clinical efficacy rate of the patients after treatment (90.74%) was significantly higher than that of the control group (75.93%).

**Table 1:** Clinical efficacy between the control and Tripterygium glycosides groups [n (%)].

Treatment	Treatment Outcome [n (%)]				
	Ineffective	Effective	Significant effect	Recovered	Total effective rate
*Tripterygium wilfordii*<br>Polysaccharide	5 (9.26)	6 (11.11)	17 (31.48)	26 (48.15)	49 (90.74)
Control	13 (24.07)	11 (20.37)	19 (35.19)	11 (20.37)	41 (75.93)
χ^2^					4.267
* P *					0.039

### Serum sex hormone levels between the control and Tripterygium glycosides groups

Before treatment, there was no statistically significant difference in serum sex hormone levels between the two groups (P>0.05). The E2 and FSH levels in both groups decreased following treatment compared with their pre-treatment levels (P<0.01). These levels were reduced than in the control group (P<0.01). Table 2

**Table 2:** Serum sex hormone levels between the control and Tripterygium glycosides groups (*x̄±s, n*=54).

Treatment	E2 (pmol/L)		FSH (U/L)	
	Before treatment	After treatment	Before treatment	After treatment
*Tripterygium wilfordii*<br>Polysaccharide	12.36±2.38	6.01±1.12	67.28±4.17	70.78±4.01
Control	12.87±2.28	9.26±1.61	68.03±5.11	50.16±4.16
T	1.137	12.177	0.836	26.224
P	0.258	<0.001	0.405	<0.001

### Tumour marker levels between the control and Tripterygium glycosides groups

The data presented in Table 3 reveal no difference in the levels of tumour markers between the two groups before treatment (P>0.05). After treatment, the CA125 and CA199 in both groups decreased than before treatment (P<0.01).

**Table 3:** Tumour marker levels between the control and Tripterygium glycosides groups (*x̄±s, n*=54).

Treatment	CA125 (kIU/mL)		CA199 (kIU/mL)	
	Before treatment	After treatment	Before treatment	After treatment
*Tripterygium wilfordii*<br>Polysaccharide	39.53±6.47	13.81±4.79	57.10±6.36	14.49±2.37
Control	39.26±6.72	18.32±5.18	57.56±6.18	22.06±3.12
* T *	0.213	4.697	0.381	14.198
* P *	0.832	<0.001	0.704	<0.001

### The levels of pro-inflammatory cytokines between the control and Tripterygium glycosides groups

We found that in the treated groups, IL-6, TNF-α, and HS-CRP levels were reduced than before treatment (P<0.01) (Table 4).

**Table 4:** The levels of pro-inflammatory cytokines between the control and Tripterygium glycosides groups (*x̄±s, n*=54).

Treatment	IL-6 (pg/mL)		TNF-α (pg/mL)		HS-CRP (mg/L)	
	Before treatment	After treatment	Before treatment	After treatment	Before treatment	After treatment
*Tripterygium*<br>*glycosides*<br>Polysaccharide	5.89±1.11	1.79±0.15	46.02±5.05	5.17±1.01	9.89±2.11	3.06±0.89
Control group	5.79±1.05	3.68±0.52	46.08±6.26	18.06±1.21	10.64±3.01	6.28±1.25
T	0.481	25.663	0.055	60.098	1.499	15.420
P	0.632	<0.001	0.956	<0.001	0.137	<0.001

### Comparison of adverse reaction incidences between the control and Tripterygium glycosides groups

An overall incidence of 7.41% (4/54 cases) of adverse reactions was observed in the control group during the treatment period, with three cases of nausea and vomiting and one case of dizziness. A total of 3.70% (2/54 cases) of adverse reactions occurred in the treatment group, including one case of nausea and vomiting and one case of dizziness.

## Discussion

EMs is a common and frequently occurring disease in gynaecology and one of the major factors contributing to infertility in women of childbearing age. The disease has a long course, which can affect the function of the visceral organs, meridians, qi, and blood to varying degrees, aggravate pelvic congestion, hinder the circulation of qi and blood, and seriously affect patients’ quality of life [7]. Currently, the clinical treatment of EMs involves pharmacotherapy. *Tripterygium* glycosides, derived from the traditional Chinese medicine *Tripterygium wilfordii*, exhibit immunosuppressive and anti-inflammatory properties. However, their toxic components have been eliminated in clinical applications. Some studies have demonstrated that *Tripterygium* glycosides can effectively prevent follicular formation by inhibiting the overexpression of inflammatory cytokine IL-6.

Furthermore, they can inhibit the growth of the ectopic endometrium by suppressing the endometrium in patients [8] [9]. Previous research has confirmed that *Tripterygium* glycosides have advantages over Western medicine in the long-term treatment of Ems [9]. Gestrinone is a synthetic steroid hormone with anti-pregestational and anti-estrogenic properties. As its primary mechanism of action, it can regulate ovarian endocrine estrogen, promote atrophy and degeneration of the ectopic endometrium, and reduce gonadotropin secretion in the hypothalamic and pituitary axes. This study used Tripterygium glycosides combined with gestrinone to treat patients with EMs. The results indicated that the total clinical efficacy rate after treatment (90.74%) was raised than that in the control group (75.93%; P<0.05), suggesting that the combined medication has improved clinical efficacy and higher safety.

E2 is secreted by follicular granulosa cells in the ovaries and can promote nerve growth in lesions and increase hyperalgesia in EMs. FSH is a regulatory glycoprotein secreted by the pituitary gland that promotes follicular maturation. E2 is the most potent estrogen secreted by the ovaries and, combined with FSH, promotes follicular development, ovulation, uterine development, and cyclic changes in the endometrium [10]. Post-treatment E2 and FSH levels were reduced than those in the control group (P<0.05), implying that combining *Tripterygium* glycosides and gestrinone can improve ovarian function in patients. CA125 and CA199 are commonly used in clinical practice to evaluate EM recurrence; elevated levels of these markers are closely associated with disease severity and recurrence [11]. Some studies have demonstrated that the serum CA125 levels in patients with EMs are higher than in the normal population. This suggests that changes in CA125 levels can be biomarkers to monitor Ems [12]. The CA125 and CA199 after treatment were reduced than those in the control group (P<0.01), implying that *Tripterygium* glycosides can improve tumour markers in patients and reduce the risk of recurrence.

IL-6 is a multifunctional inflammatory cytokine that mediates inflammation progression through humoral immunity. Its high expression can increase pelvic and fallopian tube adhesions and fibrosis. TNF-α has multiple biological functions that can initiate and promote the inflammatory immune response. Appropriate levels of TNF-α can regulate ovarian endocrine function in patients, help maintain normal ovulation and regular menstrual cycle, and encourage early embryo implantation and follicular development [13]. Abnormally elevated TNF-α levels can cause pelvic adhesions, leading to diseases such as miscarriages and infertility. HS-CRP is a common inflammatory and immune regulatory factor significantly increases blood concentration after tissue injury or acute infection. IL-6 promotes HS-CRP secretion, resulting in immune dysfunction and aggravation of inflammatory responses [14]. Th IL-6, TNF-α, and HS-CRP levels were reduced in the treatment group than in the control group after treatment (P<0.01), implying that *Tripterygium* glycosides could significantly reduce the inflammatory response in patients.

In summary, *Tripterygium* glycosides exhibit significant clinical efficacy in treating patients with EMs by significantly improving serum sex hormone levels, reducing tumour marker levels, alleviating inflammatory reactions, and exerting minimal toxic side effects.

## Dodatak

### Assurance of the originality of data

The author(s) assure the readers and the publishers that all data presented here are original.

### Authors contribution

Jianping Qiu designed and performed the experiments. Ying Xu analyzed and interpreted the data. Fangdan Xu prepared the manuscript with contributions from all co-authors.

### Data availability

The authors declare that all data supporting this study’s findings are available within the paper, and any raw data can be obtained from the corresponding author upon request.

### Conflict of interest statement

All the authors declare that they have no conflict of interest in this work.

## Dodatak

### List of abbreviations

EMs, Endometriosis;<br>CA125, Cancer antigen 125;<br>CA199, Cancer antigen 199;<br>E2, Estradiol;<br>FSH, Follicle-stimulating hormone;<br>HS-CRP, High-sensitivity C-reactive protein;<br>IL-6, Interleukin-6;<br>TNF-a, Tumour necrosis factor-alpha.
